# Effects of Microbubble Size on Ultrasound-Mediated Gene Transfection in Auditory Cells

**DOI:** 10.1155/2014/840852

**Published:** 2014-08-31

**Authors:** Ai-Ho Liao, Yi-Lei Hsieh, Hsin-Chiao Ho, Hang-Kang Chen, Yi-Chun Lin, Cheng-Ping Shih, Hsin-Chien Chen, Chao-Yin Kuo, Ying-Jui Lu, Chih-Hung Wang

**Affiliations:** ^1^Graduate Institute of Biomedical Engineering, National Taiwan University of Science and Technology, TR-916, Section 4, 43 Keelung Road, Taipei 10607, Taiwan; ^2^Graduate Institute of Medical Sciences, National Defense Medical Center, No. 161, Section 6, Minquan East Road, Taipei 114, Taiwan; ^3^Department of Otolaryngology-Head and Neck Surgery, Tri-Service General Hospital, National Defense Medical Center, No. 325, Section 2, Cheng-Kung Road, Taipei 114, Taiwan

## Abstract

Gene therapy for sensorineural hearing loss has recently been used to insert genes encoding functional proteins to preserve, protect, or even regenerate hair cells in the inner ear. Our previous study demonstrated a microbubble- (MB-)facilitated ultrasound (US) technique for delivering therapeutic medication to the inner ear. The present study investigated whether MB-US techniques help to enhance the efficiency of gene transfection by means of cationic liposomes on HEI-OC1 auditory cells and whether MBs of different sizes affect such efficiency. Our results demonstrated that the size of MBs was proportional to the concentration of albumin or dextrose. At a constant US power density, using 0.66, 1.32, and 2.83 *μ*m albumin-shelled MBs increased the transfection rate as compared to the control by 30.6%, 54.1%, and 84.7%, respectively; likewise, using 1.39, 2.12, and 3.47 *μ*m albumin-dextrose-shelled MBs increased the transfection rates by 15.9%, 34.3%, and 82.7%, respectively. The results indicate that MB-US is an effective technique to facilitate gene transfer on auditory cells *in vitro*. Such size-dependent MB oscillation behavior in the presence of US plays a role in enhancing gene transfer, and by manipulating the concentration of albumin or dextrose, MBs of different sizes can be produced.

## 1. Introduction

Mammalian cochlear hair cells do not regenerate naturally, and hence damage to them can result in permanent hearing loss. This has prompted considerable attention to be paid to the regeneration of inner ear hair cells, which has led to demonstrations of the feasibility of gene therapy and stem cell transplantation for inner ear disease [1, 2].

Gene therapeutic approaches to several forms of hearing disorders have been experimentally investigated using either viral [3, 4] or nonviral [5] vectors. Delivery methods mainly utilize two surgical routes: direct injection or diffusion through the round window membrane (RWM) [5] or intracochlear infusion through a cochleostomy or canalostomy on the semicircular canal [4, 6, 7]. Although these deliveries achieve different transfection efficiency, the associated surgical trauma, inflammation, and possible damage in hearing deterioration suggest that a less invasive delivery method for future clinical application is imperative. Our previous study demonstrated the practical application of combining microbubbles (MBs) with ultrasound (US) to increase the RWM permeability for facilitating drug or medication delivery to the inner ear in an animal model [8]. Before extending this minimal invasive technique for application of inner ear gene therapy* in vivo* (unpublished data), the present study focuses on evaluating the impact of the MB-US technique for improving the efficacy of gene transfection on auditory cells—the HEI-OC1 cells—and the difference by manipulating different-sized MBs in transfection* in vitro*.

US-targeted MB destruction is a safe and noninvasive gene transfection technology. MBs are known to be contrast agents for ultrasonic imaging (their primary medical use). Recently, additional medical applications for MBs have focused on utilizing the interaction of MBs and US to produce microstreaming, known as cavitation. MBs are well suited as carriers of genes and drugs. Compared with viral vectors, MBs have greater capacity and can carry antisense oligonucleotides, DNA fragments, and even the entire chromosome [9]. The energy in the US field destroys the MBs, with cavitation or other mechanical effects increasing both the cell membrane permeability and the dimensions of the intercellular space between endothelial cells; these effects can lead to the drug or gene more easily reaching the tissue or cells via the rupture site and the widened interstitial space [10–13].

Microbubbles in various sizes have different effects on resonance frequency, expansion ratio, pressure thresholds for inertial cavitation and fragmentation, translational velocity, and lifetime of stable oscillation of MBs [14–17]. Although previous study has revealed an association between the focused US-induced blood brain barrier (BBB) opening and MB size [18], most of these investigations focused on lipid-shelled MBs. In addition, dextrose was found to be able to change the characteristics of the MB shell, and increased dextrose concentration could produce MBs with thinner shells, good stability, and a wider range of resonance frequency, thus enhancing the efficiency of gene transfection [19]. However, the effects of albumin and dextrose concentrations on the MB size dependence of US-induced gene transfection remain unclear. Moreover, cell membranes in different cell types have different biophysical characteristics in terms of transfection and physical stability [20].

The present study aimed to evaluate the impact of the MB-US technique on gene transfection efficiency in auditory cells and to explore the ratio of glucose and albumin concentrations for producing albumin-shelled MBs in different sizes to improve the transfection rate* in vitro*.

## 2. Material and Methods

### 2.1. Preparation of Albumin-Shelled MBs

**Figure 1:**
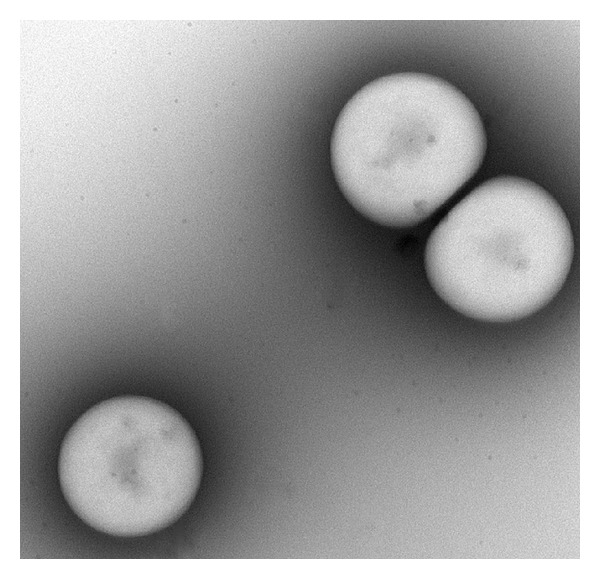
TEM image of albumin-shelled MBs.

Albumin MBs were prepared according to the procedure used in our previous studies [8, 21, 22].  Figure 1 illustrates the TEM image of albumin MBs. The shell thickness of the albumin MBs was about 30–60 nm [23]. For albumin/dextrose MB preparation, dextrose (D(+)-glucose; Acros Organics, Fair Lawn, NJ, USA) was purchased to prepare stock solutions of 5%, 10%, 15%, 20%, 30%, 40%, and 45% (weight (w)/volume (v)) dextrose in physiological saline (pH 7.4, 0.9% sodium chloride). Human serum albumin (HSA) was purchased as a sterile 20% solution (Octapharma, Vienna, Austria), which was diluted with physiological saline to make stock solutions containing 0.66%, 1.32%, 2%, 3.5%, or 5% (w/v) HSA. Briefly, albumin/dextrose MBs were generated by sonication in 10 mL of solution by mixing the albumin and dextrose (Table 1) with perfluorocarbon gas in physiological saline using a sonicator (Branson Ultrasonics, Danbury, CT, USA) for 2 min. The number of perfluorocarbon-filled albumin-(Gd-DTPA) MBs in the solution was measured with an electrical sensing zone (ESZ) device (MultiSizer III; Beckman Coulter, Fullerton, CA, USA) using a 30 *μ*m-aperture probe with measurement boundaries of 0.6–20 *μ*m. The size distribution in the suspension was measured based on dynamic light scattering (Zetasizer Nano ZS90; Malvern Instruments, Worcestershire, UK).

### 2.2. Measuring the Concentrations of MBs of Different Sizes after US Treatment

A 2% agarose phantom was constructed with a 2 × 2 × 20 mm^3^ chamber at its center to load the albumin MBs of different sizes. The loaded phantom was then sonicated by a 440 kHz US transducer (KTAC-4000; NepaGene, Chiba, Japan) at an acoustic pressure of 2.5 W/cm^2^ for 1 min. The burst rate was 20 Hz, the duty cycle was 50%, and a 2 mm-diameter transducer was used. After the MBs had been destroyed by the US excitation, a spectrophotometric method was used to measure the MB concentration. MB suspensions were diluted in phosphate-buffered saline (PBS) to an estimated optical density at 530 nm (OD530) of between 0.3 and 2.4. The OD530 was then measured, and the MB concentration was determined using previously established standard curves of OD530 versus the MB concentration (measured using an ESZ device). Calibration curves were established for different MB concentrations because the amount of light scattered (and hence optical density) increases with increasing MB concentration [24]. The concentration change was calculated as the percentage decrease in the optical density as follows:

(1) ΔOptical  density  at 530 nm(%)  =ODpre−ODpostODpre×100%,

where OD_pre_ and OD_post_ are the optical densities of the MBs before and after US treatment, respectively.

### 2.3. Plasmid DNA

The 4.2 kbp pEGFP-C1 reporter plasmid (Roche Applied Science, Rotkreuz, Switzerland), encoding the enhanced green fluorescent protein gene, was amplified in* Escherichia coli* and purified using the EndoFree Plasmid Mega Kit (Qiagen, Valencia, CA, USA) in accordance with the manufacturer's user manual. The concentration of pEGFP was measured using a hybrid multimode microplate reader (Synergy H4; BioTek, Winooski, VT, USA).

### 2.4. Cell Culture

HEI-OC1 cells, an auditory hair cell line that retains cell division activity [25], were kindly donated by Dr. Federico Kalinec (House Ear Institute, Los Angeles, CA, USA). HEI-OC1 cells were cultured in Dulbecco's modified Eagle's medium (DMEM) (Gibco, Grand Island, NY, USA) containing 10% fetal bovine serum (FBS; Biological Industries, Kibbutz Beit-Haemek, Israel) without antibiotics under permissive conditions (33°C and 10% CO_2_).

### 2.5. Ultrasound-Mediated Gene Transfer In Vitro

The experiments were divided into three parts. The first part was designed to improve the transfection efficiency by combining US with MBs. The experiments were randomly divided into the following five groups with different experimental parameters (*n* = 5 per group): (1) control, (2) plasmid only (P), (3) US combined with plasmid (U), (4) MBs in a short time soaking up the cells and combined with plasmid (M), and (5) US combined with MB and plasmid (UM). The second and third parts involved evaluating the transfection efficiency when using albumin- or albumin-dextrose-shelled MBs of three different sizes. For this, the experiments were randomly divided into four groups with different experimental parameters (*n* = 5 per group): (1) control, (2) plasmid only, (3) plasmid combined with MBs of three different sizes, and (4) US combined with MBs of three different sizes and with plasmid.

HEI-OC1 cells were seeded at 4 × 10^4^ cells/well onto a 24-well cell culture plate and grown overnight. pEGFP-C1 (0.25 *μ*g) and 1.125 *μ*L of Lipofectamine reagent (FuGENE HD Transfection Reagent; Roche, Mannheim, Germany) were diluted in 20 *μ*L of serum-free DMEM medium for 15 min according to the manufacturer's instructions to allow pEGFP-C1-Lipofectamine complexes to form.

**Figure 2:**
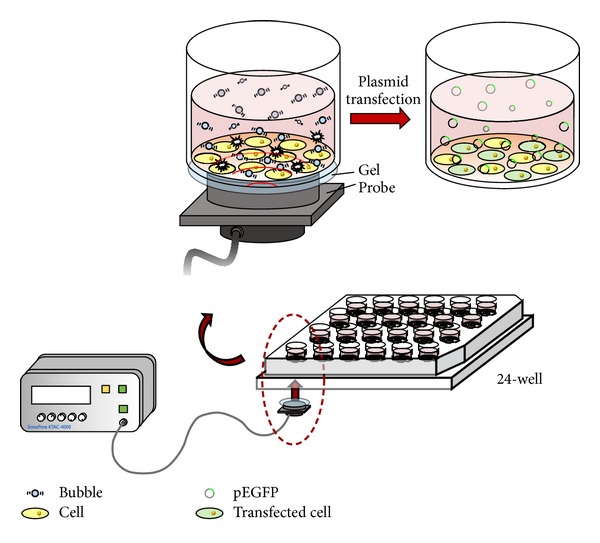
Experimental setup for ultrasound-mediated gene transfer* in vitro*.

Shortly before transfection, the serum-containing medium was removed from the cell culture plate and replaced with 4 × 10^7^/200 *μ*L MBs. As shown in Figure 2, the plate was sonicated by US from the bottom to the top for 2 min before being rinsed with PBS three times. The MBs were then replaced by 400 *μ*L of 10% serum-containing medium. The pEGFP-C1-Lipofectamine complex was added, and the sample was placed in the incubator for 24 h. The mode of US was set as follows: voltage of 30 V, transducer with a center frequency of 3.185 MHz, duty cycle of 50%, burst rate of 2 Hz, acoustic intensity of 0.46 W/cm^2^, and 2 min duration. The medium was exchanged for DMEM, and the cells were incubated for another 24 h. The transfection efficiency was determined with the aid of fluorescence microscopy (CKX41; Olympus, Tokyo, Japan). Cell lysate buffer (radioimmunoprecipitation assay, RIPA) was then added for cell lysis. The fluorescence intensity of green fluorescent protein was measured using a hybrid multimode microplate reader. The normalized fluorescence increase was calculated as follows:

(2) Normalized  fluorescence  increase  (%)  =FIUS+MB−FIMBFIMB×100%,

where FI_US+MB_ and FI_MB_ are the fluorescence intensities in groups UM and M, respectively.

### 2.6. Cell Viability for Different US Power Densities

Cells were seeded at 3 × 10^5^ cells/well onto a 24-well cell culture plate and grown for 24 h. The culture medium was then replaced with 4 × 10^7^/200 *μ*L MBs with a mean diameter of 1.32 *μ*m and sonicated by US at different power densities (0.2, 0.46, and 0.84 W/cm^2^) for 2 min. The well plate was then rinsed with PBS three times, replaced by 10% FBS culture medium, and cultured for 24 h. In accordance with the manufacturer's protocol, cell proliferation reagent (WST-1; Roche) was added at 16 *μ*L/well and cultured for 4 h. The survival rate was quantified based on the 450 nm absorbance values measured using a hybrid multimode microplate reader.

### 2.7. Statistical Analysis

The obtained data were analyzed statistically using Student's *t*-test and results are expressed as means ± standard deviation (SD). Differences were considered significant at *P* < 0.05.

## 3. Results

### 3.1. Production of Albumin-Dextrose-Shelled MBs of Different Sizes

**Figure 3:**
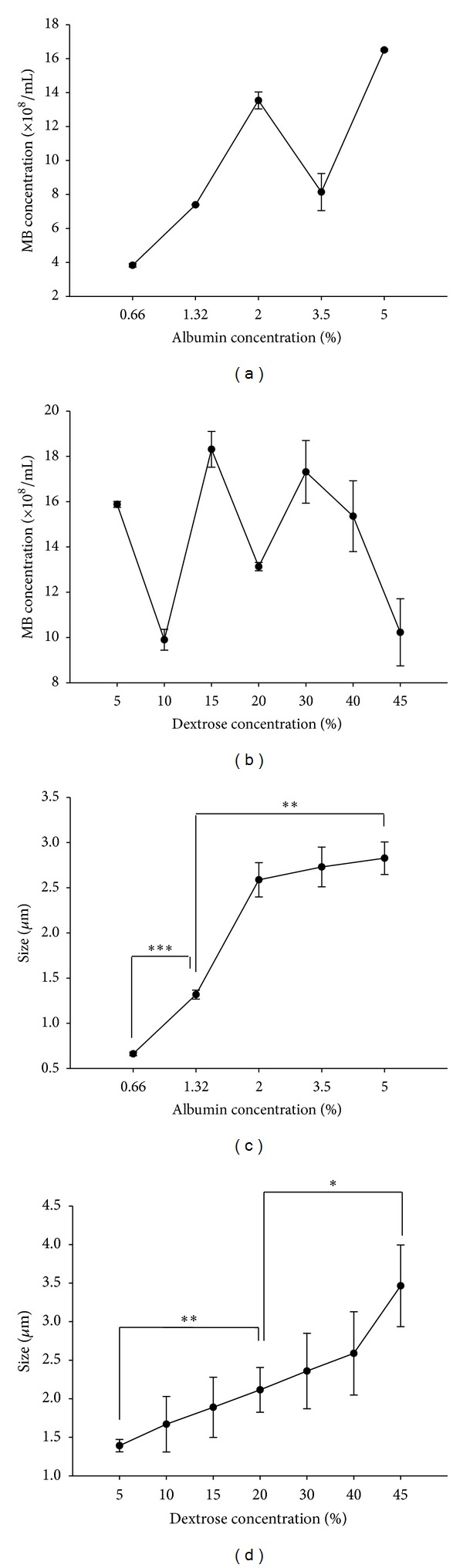
Production of albumin-dextrose-shelled MBs of different sizes. (a) Relationships between MB concentration and albumin concentration, (b) MB concentration and dextrose concentration, (c) MB size and albumin concentration, and (d) MB size and dextrose concentration. Results are expressed as mean ± standard deviation (*n* = 5). *indicates *P* < 0.05; **indicates *P* < 0.01; ***indicates *P* < 0.005.

The concentration of the produced MBs was not proportional to the albumin concentration (Figure 3(a)) or the dextrose concentration (Figure 3(b)), but their size was influenced by changing the composition ratio of albumin alone or of dextrose in the albumin (Figures 3(c) and 3(d)). When the compositions of albumin in saline were 0.66%, 1.32%, 2%, 3.5%, and 5%, the produced MBs had diameters (means ± SD) of 0.66 ± 0.02, 1.32 ± 0.05, 2.59 ± 0.19, 2.73 ± 0.22, and 2.83 ± 0.18 *μ*m, respectively (Figure 3(c)). When the compositions of dextrose in the 1.32% albumin saline solution were 5%, 10%, 15%, 20%, 30%, 40%, and 45%, the produced MBs had diameters of 1.39 ± 0.08, 1.67 ± 0.36, 1.89 ± 0.39, 2.12 ± 0.29, 2.36 ± 0.49, 2.59 ± 0.54, and 3.47 ± 0.53 *μ*m, respectively (Figure 3(d)). The conditions of different compositions of albumin and dextrose in producing MBs of different sizes are summarized in Table 1. Increasing the albumin concentration from 0.66% to 5% increased the albumin MB size 3.3-fold, while increasing the dextrose concentration from 5% to 45% in 1.32% albumin composition increased the albumin/dextrose MB size 2.1-fold. These results indicate that the size of produced MBs could be proportional either to the albumin concentration alone in preparing albumin MBs or to the dextrose concentration in 1.32% albumin composition in preparing albumin/dextrose MBs. Furthermore, for both high albumin (5%) and high dextrose (45%) concentrations and both low albumin (0.66%) and low dextrose (10%) concentrations, the MB sizes were 1.66 ± 0.13 and 1.33 ± 0.24 *μ*m, respectively. At a low dextrose concentration (10%) composition, the increased MB size would be dependent on the increased albumin concentration, whereas at a high dextrose concentration (45%), the high albumin concentration conversely produced small size MBs (Table 1).

### 3.2. Destruction Efficiency for MBs of Different Sizes

**Figure 4:**
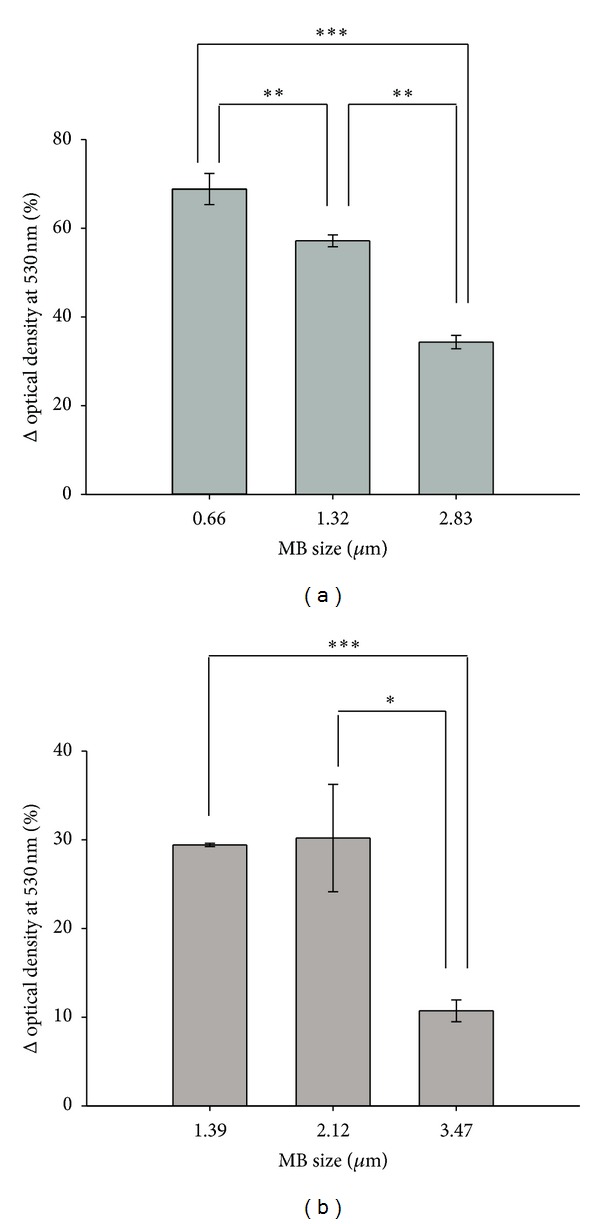
Destruction efficiency for MBs of different sizes. (a) Destruction efficiencies of albumin MBs and (b) albumin/dextrose MBs of different sizes. Results are expressed as mean ± standard deviation with *n* = 5 for each bar. *indicates *P* < 0.05; **indicates *P* < 0.01; ***indicates *P* < 0.005.

To investigate whether destruction efficiency is dependent on the size of MBs, a phantom loaded with different-sized MBs was sonicated by a 440 kHz US transducer at an acoustic pressure of 2.5 W/cm^2^ for 1 minute. The results showed that the destruction efficiencies of albumin MBs with diameters of 0.66, 1.32, and 2.83 *μ*m were 68.84 ± 3.52%, 57.16 ± 1.33%, and 34.33 ± 1.52%, respectively (Figure 4(a)); while for albumin/dextrose MBs with diameters of 1.39, 2.12, and 3.47 *μ*m, they were 29.42 ± 0.19%, 30.2 ± 6.05%, and 10.72 ± 1.24%, respectively (Figure 4(b)). Results of the destruction efficiency of MBs of different sizes suggest that MBs smaller than 2 *μ*m were easier to destroy for a constant US power density (Table 2).

### 3.3. Transfection Efficiency for MBs of Different Sizes

**Figure 5:**
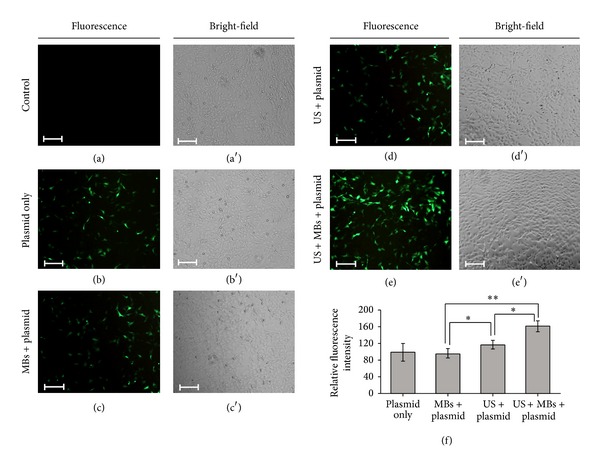
The transfection efficiency of HEI-OC1 cells with plasmid DNA by using a combination of US and MBs. (a, a′) Fluorescence and bright-field images of living cells in the control groups, (b, b′) plasmid only, (c, c′) MBs with plasmid, (d, d′) US with plasmid, and (e, e′) US with MBs and plasmid. (f) Fluorescence intensities in the five groups quantified and normalized relative to the control group. Scale bar = 200 *μ*m. Results are expressed as mean ± standard deviation with *n* = 5 for each bar. *indicates *P* < 0.05; **indicates *P* < 0.01.

The transfection efficiency of HEI-OC1 cells with plasmid DNA by using a combination of US and MBs was investigated (Figure 5). The fluorescence intensities (in arbitrary units) in groups of P, M, U, and UM were 796.3 ± 211.9, 774.3 ± 125.1, 978.3 ± 101.0, and 1270.3 ± 146.7, respectively; the transfection efficiency was highest in group UM and was significantly higher in groups UM (*P* < 0.01) and U (*P* < 0.05) than in control group P. There was no significant difference between groups P and M, indicating that MBs would not influence the transfection efficiency in the short time that they soak up the cells without the US treatment.

**Figure 6:**
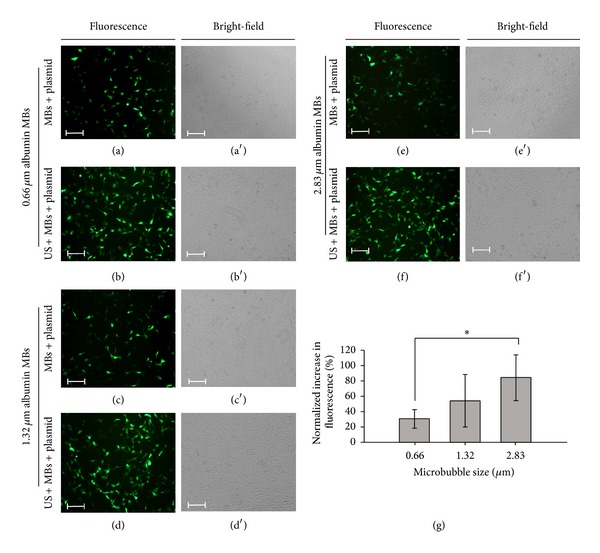
The transfection efficiency when using albumin MBs of three different sizes. (a, a′) Fluorescence and bright-field images of living cells in the groups of 0.66 *μ*m albumin MBs combined with plasmid, (b, b′) US combined with 0.66 *μ*m albumin MBs and plasmid, (c, c′) 1.32 *μ*m albumin MBs combined with plasmid, (d, d′) US combined with 1.32 *μ*m albumin MBs and plasmid, (e, e′) 2.83 *μ*m albumin MBs combined with plasmid, and (f, f′) US combined with 2.83 *μ*m albumin MBs and plasmid. (g) Fluorescence intensities for MBs of three sizes quantified and normalized relative to the control group. Scale bar = 200 *μ*m. Results are expressed as mean ± standard deviation with *n* = 5 for each bar. *indicates *P* < 0.05.

The transfection efficiency when using albumin MBs of three different sizes was also evaluated (Figure 6). The normalized percentage increases in fluorescence for US and plasmid combined with 0.66, 1.32, and 2.83 *μ*m MBs were 30.58 ± 12.53%, 54.10 ± 33.95%, and 84.74 ± 29.37%, respectively; the transfection efficiency showed an MB size-dependent tendency and was highest for US combined with 2.83 *μ*m MBs and plasmid. In comparison, the transfection efficiency of 2.83 *μ*m MBs was significantly higher than that of 0.66 *μ*m MBs (*P* < 0.05), whereas there was no significant difference between 0.66 and 1.32 *μ*m MBs. Taken together, the results indicate that the transfection efficiency could be improved by 54.16% when the size of the albumin MBs is increased 3.3-fold.

**Figure 7:**
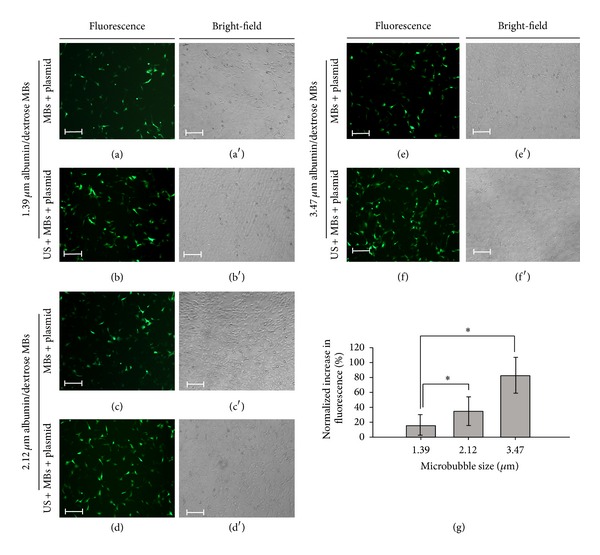
Transfection results for HEI-OC1 cells with albumin/dextrose MBs of three different sizes. (a, a′) Fluorescence and bright-field images of living cells in the groups of 1.39 *μ*m albumin/dextrose MBs combined with plasmid, (b, b′) US combined with 1.39 *μ*m albumin/dextrose MBs and plasmid, (c, c′) 2.12 *μ*m albumin/dextrose MBs combined with plasmid, (d, d′) US combined with 2.12 *μ*m albumin/dextrose MBs and plasmid, (e, e′) 3.47 *μ*m albumin/dextrose MBs combined with plasmid, and (f, f′) US combined with 3.47 *μ*m albumin/dextrose MBs and plasmid. (g) Fluorescence intensities for MBs of three sizes quantified and normalized relative to the control group. Scale bar = 200 *μ*m. Results are expressed as mean ± standard deviation with *n* = 5 for each bar. *indicates *P* < 0.05.

Transfection results for HEI-OC1 cells with albumin/dextrose MBs of three different sizes are shown in Figure 7. The normalized percentage increases in fluorescence for US and plasmid combined with 1.39, 2.12, and 3.47 *μ*m MBs were 15.86 ± 13.56%, 34.34 ± 19.98%, and 82.67 ± 24.47%, respectively. The transfection efficiency differed significantly between these three groups, with improving efficiency as the size of the albumin/dextrose MBs increased.

### 3.4. Cell Viability Analysis

**Figure 8:**
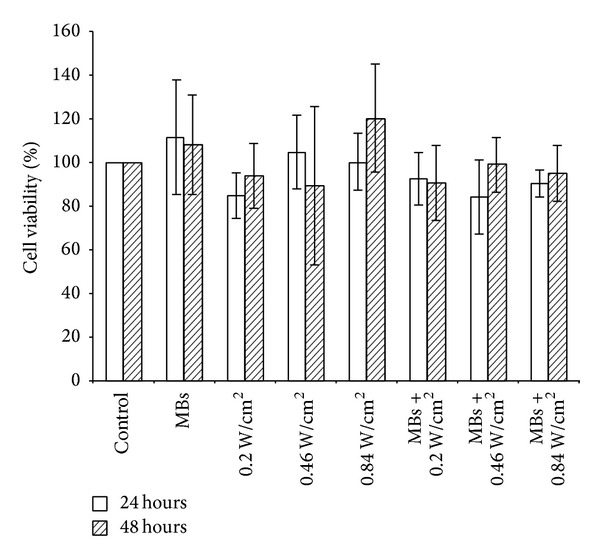
Cell survival rates in the different groups at 24 and 48 hours.

To investigate whether the application of US and MBs would cause cell damage, cell viability following MBs and US treatments with different power densities was tested. There were no statistically significant differences among the various groups (*P* > 0.05) by quantifying the 24 and 48 h cell survival (Figure 8), confirming that using US at power densities from 0.2 to 0.84 W/cm^2^ did not affect the survival of HEI-OC1 cells even when combined with MBs.

## 4. Discussion

Although previous investigators have depicted protocols for producing uniformly sized MBs [24], methods that often involve several preparation procedures and parameter settings, in this study we produced different-sized MBs through a simple laboratory method by adjusting the concentration of albumin and dextrose. We found that the MB would increase in size as the proportion of albumin or dextrose in the composition increased. In the albumin and dextrose mixed condition, a larger MB was produced under two conditions: (1) a higher dextrose concentration and a lower albumin concentration and (2) a lower dextrose concentration (or without adding glucose) and a higher albumin concentration. This report describes the first successful demonstration of the correlation of albumin, dextrose, and albumin-dextrose compositions in producing MBs of different sizes.

The efficiency of gene transfer was found to increase with increasing MB diameter. Although the mechanism for MB size-dependent sonoporation increase is not yet known, the duration of the MB's interaction with the vessel wall has been found to increase with increasing MB diameter [14]. Using focused US in conjunction with MBs, the exchange rate between the blood plasma and the brain tissue has been shown to be proportional to the MB size [26]. The opening volume of the BBB in the mouse brain using small bubbles was found to be significantly lower than for the larger MBs [27], which is in agreement with our findings that gene transfer efficiency is dependent on the MB size.

MBs produced in this study had sizes ranging from 0.5 to 3.5 *μ*m. The efficiency of MB destruction appears to be conversely proportional to the MB size; larger MBs were more resistant to destruction, whereas in gene transfer, larger MBs exhibited more enhanced transfection efficiency because MB behavior depends not only on the US parameters but also on the MB size and physicochemical properties. Various MB dynamic activities generated by US exposure include local fluid microstreaming, shear stress, and high-speed fluid microjet, leading to different sonoporation effects [28–30]. In this experiment, MBs smaller than 2 *μ*m were easier to destroy for a constant US power density, implying that oscillation of small-size MBs may become unstable under US activation and may fragment prior to interacting with cells. Such effects have also been noted in previous study [27]. In contrast, stable cavitation within the lifetime of the larger MB may play a role in increasing sonoporation of gene transfer on cultivated HEI-OC1 cells. In addition, an MB's motion and linear oscillation when in close contact with the cell membrane can cause local deformation and transient porosity in the cell membrane without rupturing it [31]. Therefore, the efficiency of MB destruction may not faithfully reflect the efficacy of gene transfer, as shown in our data.

Our experiments also demonstrated that gene transfer assisted by 2.83 *μ*m albumin MBs-mediated US was able to achieve a gene transfection efficiency of around 85%, which was significantly higher than those in the other treatment groups and was shown without adverse effect on cellular viability. This transfection efficiency is higher than the previously reported value of 70% when using 2.89 or 2.98 *μ*m lipid MBs [32], suggesting that different MB shell compositions, such as surfactants, lipids, proteins, polymers, or a combination of these materials, may consist of different physicochemical properties for a wide variety of biomedical applications. For gene transfer applications, albumin-based MBs can potentially incorporate large amounts of plasmid DNA within the thick protein shell, whereas the drug-carrying capacity of lipid-based MBs is relatively low [33]. Due to covalent cross-linking, albumin MBs form relatively rigid shells by disulfide bridging of proteins as compared with other compositions of MBs [34]; this stabilizes the shell and prevents gas dissolution, resulting in a greater yield and increasing their acoustic durability.

Compared to conventional liposome-mediated transfection, our results demonstrated that MB-US-mediated gene transfer appears to be advantageous for increasing the cell membrane permeability of auditory cells and allowing plasmid DNA to enter the cells. Additionally, the proximity between the MB, nucleic acid, and target cells would be expected to enhance cell “poration” effects, thus improving nucleic acid transfer to the target cell [35–37]. To facilitate the efficiency of gene transfer, some previous studies proposed using cationic MBs to augment interactions between MBs and cells and reduce the separation between MBs and plasmid DNA [32, 37]. Cationic MBs (zeta potential of approximately 4-5 mV relative to the surrounding environment) were found to be attracted to negatively charged plasmid, while the neutral MBs (−0.7 mV) did not attract plasmid. Those studies demonstrated the influence of MB surface modifications on their interaction with plasmid DNA and target cells and the functional consequences of those interactions in terms of US-mediated gene transfer [37]. In the present study, the albumin-shelled MBs were negatively charged (−21.4 mV) [19, 38], with this negative charge increasing the binding of positively charged liposomes to the cells and influencing the transfection efficiency.

## 5. Conclusion

This study explored the impact of MB size-dependent gene transfer* in vivo*. Larger MBs exhibited an increased resistance to ultrasonic destruction and enhanced the transfection efficiency of auditory hair cells for a constant US power density. Production of MBs in different sizes can be manipulated by adjusting the concentration of albumin or dextrose alone or the combined albumin and dextrose mixture. This study provides a promising strategy for auditory cell gene transfer* in vivo* by using MB application in the presence of US. The recommended safe power densities would be from 0.2 to 0.84 W/cm^2^. Further research is needed to clarify the feasibility of using MB-US as a tool to improve inner ear gene transfer* in vivo*.

## Figures and Tables

**Table 1:** Summary of different compositions of albumin and dextrose in producing MBs of different sizes.

MB diameter (*μ*m)	Albumin conc. (w/w) (0% dextrose)	Dextrose conc. (w/w) (1.32% albumin)	Albumin conc./ dextrose conc. (w/w)
<1.0	0.66	NM	NM
1.0–1.5	1.32	5	0.66/10
1.5–2.0	NM	10, 15	5/45
2.0–2.5	NM	20, 30	2/10, 3.5/45, 3.5/10, 2/45
2.5–3.0	2, 3.5, 5	40	5/10
3.0–3.5	NM	45	0.66/45

Conc.: concentration; w/w: percentage weight/weight; NM: not measured.

**Table 2:** Destruction efficiency of MBs of different sizes.

ΔOptical density at 530 nm (%)	Sample		
	Increased albumin conc. (0% dextrose)	Increased dextrose conc. (1.32% albumin)	Adjusted albumin conc. & dextrose conc.
>60	<1.0 *μ*m	NA	NA
50–60	1.0–1.5 *μ*m	1.5–2.0 *μ*m	NA
40–50	NA	1.5–2.0 *μ*m	NA
30–40	2.5–3.0 *μ*m	2.0–2.5 *μ*m	2.0–2.5 *μ*m
20–30	2.5–3.0 *μ*m	1.0–1.5 *μ*m	2.0–3.5 *μ*m
10–20	2.5–3.0 *μ*m	2.0–3.5 *μ*m	1.0–2.0 *μ*m
0–10	NA	NA	2.0–2.5 *μ*m

Conc.: concentration and NA: not applicable.
